# Further delineation of the etiology of liver abscesses in cattle and indication of hindgut as a potential source of pathogens

**DOI:** 10.1128/spectrum.02539-25

**Published:** 2025-10-08

**Authors:** Harith M. Salih, Alyssa Deters, Raghavendra G. Amachawadi, Haiyan Wang, Taghreed Mahmood, Xiaorong Shi, Mina Abbasi, Leigh Ann George, Ty E. Lawrence, T. G. Nagaraja

**Affiliations:** 1 Department of Clinical Sciences, College of Veterinary Medicine, Kansas State University 70725, Manhattan, Kansas, USA; 2 Department of Diagnostic Medicine/Pathobiology, College of Veterinary Medicine, Kansas State University 733350, Manhattan, Kansas, USA; 3 Department of Statistics, College of Arts and Sciences, Kansas State University 326938, Manhattan, Kansas, USA; 4 Beef Carcass Research Center - Department of Agricultural Sciences, Paul Engler College of Agriculture and Natural Sciences, West Texas A&M University 383821, Canyon, Texas, USA; Michigan State University, East Lansing, Michigan, USA

**Keywords:** liver abscess, feedlot cattle, major and minor bacterial pathogens, ruminal and colonic epithelial tissues, culture method, qPCR assay

## Abstract

Liver abscesses (LA) in feedlot cattle are polymicrobial infections, initiated primarily by the translocations of the two subspecies of *Fusobacterium necrophorum, necrophorum* and *funduliforme,* and secondarily by *Trueperella pyogenes* and *Salmonella enterica* from the rumen into the liver via portal blood. The hindgut has not been investigated as a potential source of LA pathogens. Our objectives were to analyze LA and matched ruminal and colonic epithelial tissues (*n* = 96), collected at a commercial beef processor immediately after evisceration, to determine culture-based prevalence of pathogens, specifically, *Escherichia coli*, *Klebsiella pneumoniae*, *Pseudomonas aeruginosa*, and *Bacteroides fragilis,* which have not been targeted before, in addition to *F. necrophorum, T. pyogenes,* and *S. enterica,* and quantitative PCR-based prevalence and concentrations of the *F. necrophorum* subspecies. The subsp. *necrophorum* was the most dominant species in LA detected by both methods, and concentrations were in the range of 7.0 log_10_ colony forming units (CFU). Fusobacterial concentrations were higher in ruminal epithelial than in colonic epithelial tissues, but colonic epithelial tissues yielded more subsp. *necrophorum* isolates than ruminal epithelial tissues. Of the targeted bacterial species, *E. coli* was the most dominant, and none of the tissues was positive for *B. fragilis*. A majority of the isolations of *E. coli* were after enrichment, suggesting the concentrations were too low to contribute to the abscess development. The data reaffirm that LA are polymicrobial infections, *F. necrophorum,* specifically *necrophorum,* is the most dominant species prevalent, and the hindgut is likely to be a source, in addition to the rumen, of pathogens that cause LA.

**IMPORTANCE:**

Liver abscesses (LA) in beef cattle are initiated by entry of bacterial pathogens from the rumen via portal blood. The two most frequently isolated pathogens are the two subspecies of *Fusobacterium necrophorum,* followed by *Trueperella pyogenes* and *Salmonella enterica*. Our objectives were to determine the prevalence of pathogens that have been reported sporadically and have not been targeted before. Liver abscesses and matched ruminal epithelium and colonic epithelial tissues, collected from feedlot cattle at slaughter, were analyzed by culture and PCR methods. The subsp. *necrophorum* was the most dominant bacterium in liver abscesses. Colonic epithelial tissues yielded more subsp. *necrophorum* than ruminal epithelial tissues. Although *Escherichia coli* was the second most prevalent species, a majority of the isolates were obtained after enrichment, indicating low concentrations. Our data reaffirm that *F. necrophorum* is the dominant species prevalent in LA, and the hindgut is likely to be another source of pathogens that cause LA.

## INTRODUCTION

Liver abscesses in cattle are a significant economic concern to the feedlot industry because of liver and offal condemnations (1), and more importantly, their impact on cattle growth performance (2) and carcass yield and attributes (3, 4). Among the bacterial pathogens implicated, *Fusobacterium necrophorum*, a ruminal bacterium, is the most frequently isolated. Two subspecies of *F. necrophorum—necrophorum* and *funduliforme*—have been identified, differing in a number of characteristics, notably prevalence in liver abscesses, likely because of differences in virulence potential. The subspecies *necrophorum* is more virulent; therefore, it is isolated more frequently (57%–100%; mean 89%) from liver abscesses compared to subsp. *funduliforme* (16%–48%; mean 28%; [5]). The second most frequently isolated pathogen is *Trueperella pyogenes*, a gram-positive facultative anaerobe, with prevalence ranging from 0 to 90%, but generally around 25%–30% of liver abscesses harbor it (1, 6, 7). Another pathogen, *Salmonella enterica,* was first reported in liver abscesses of calf-fed Holstein steers (8), but subsequently has been isolated from liver abscesses in crossbred beef cattle, culled beef cows, and beef-on-dairy cattle (1, 6, 9). In addition, a number of other bacterial species, both aerobic and anaerobic, have been isolated from liver abscesses (6, 10, 11). Culture-independent studies, based on 16S amplicon sequencing or metagenomic analyses, have further affirmed the complexity of the bacterial community composition of liver abscesses (12–15). These studies demonstrated the dominance of phylum Fusobacteriota but have consistently identified Pseudomonadota and Bacteroidota as the next two dominant phyla in purulent materials of liver abscesses. At the genus level, after *Fusobacterium*, *Pseudomonas, Bacteroides,* and *Porphyromonas* have been identified as the dominant genera (12, 15). These findings underscore the need for targeted culture-based studies to understand the prevalence of lesser-known bacterial species in liver abscess formation.

It has long been recognized that abscesses result from the translocation of pyogenic bacteria across the ruminal epithelium into the portal circulation, eventually reaching the hepatic parenchyma (10). In cattle fed high-grain diets, ruminal acidosis and subsequent rumenitis serve as major predisposing factors (5, 16–18). The chronic exposure to acidity compromises the ruminal epithelium, allowing *F. necrophorum* and *T. pyogenes* to invade and cause rumenitis. Subsequently, bacteria enter the portal circulation to reach the liver to create abscesses (10, 19, 20). While the origin of *S. enterica* in liver abscesses remains uncertain, the hindgut is suspected to be the source, given its role as the natural habitat for this pathogen (21). The hindgut, primarily the colon, contributes to approximately 5 to 10% of the total tract carbohydrate digestion in cattle (22). Dietary factors that promote increased flow of starch to the hindgut will lead to increased production of short-chain fatty acids, possibly lactic acid, resulting in hindgut acidosis (23). Although the mucous layer provides some protective barrier function, a single layer of cells is intuitively more vulnerable to chronic acidity and bacterial translocation than the stratified ruminal epithelium (5). In a serial slaughter study that tested the effects of corn stalk inclusion with and without tylosin in finishing beef steers, Jennings et al. (24) reported that 7 of 136 colonic epithelial tissues yielded *F. necrophorum* subsp. *necrophorum*. The findings highlight the need to reevaluate the traditional view of the rumen as the sole source of liver abscess pathogens.

The objectives of the study were to reevaluate the etiology of liver abscesses by focusing on lesser-known pathogens, which have not been targeted before, and to assess whether the hindgut serves as a source of pathogens that reach the liver to cause abscesses. Specifically, the objectives were to analyze liver abscesses and matched ruminal and colonic epithelial tissues, collected from feedlot cattle at slaughter, to determine (i) culture method-based prevalence of *Escherichia coli* (EC), *Klebsiella pneumoniae*, *Pseudomonas aeruginosa*, and *Bacteroides fragilis,* in addition to *F. necrophorum, T. pyogenes,* and *S. enterica*; and (ii) quantitative PCR (qPCR)-based prevalence and concentrations of the two subspecies of *F. necrophorum*.

## MATERIALS AND METHODS

### Sample collection

Liver abscesses, along with matched ruminal and colonic epithelial tissues, were collected from feedlot cattle at a federally inspected slaughter beef processing facility. A total of 96 cattle with liver abscesses, originating from 15 feedlots that did not use tylosin in their finishing diets, were sampled. From each animal, a portion of liver containing an intact abscess and approximately 5 × 10 cm sections of epithelial tissue from the ventral sac of the rumen and spiral colon were collected. Samples were placed individually in Whirl-Pak bags, stored on ice, and shipped overnight to the Anaerobe Laboratory in the College of Veterinary Medicine at Kansas State University.

### Sample processing

The abscess capsule was surface-sterilized using a red-hot spatula and incised with a sterile scalpel to access the purulent material. A sterile cotton swab was used to collect samples from the inner wall of the capsule, which were directly inoculated onto agar plates for bacterial isolation. Ruminal and colonic epithelial tissues were rinsed in RO (reverse osmosis) water to remove adherent feed particles. Ruminal papillae were cut into small pieces, and colonic mucosal layers were scraped with a sterile scalpel. Approximately 5 g of each ruminal and colonic epithelial tissues and purulent materials from liver abscesses, mixed with capsular wall tissue, were suspended in 45 mL of sterile phosphate-buffered saline. Weights of tissue samples were recorded. The liver and epithelial tissue suspensions were homogenized in a NutriBullet Pro Blender (NutriBullet, Los Angeles, CA). To ensure sterility and avoid cross-contamination, the blending cup and blades were thoroughly washed and UV-sterilized between each sample. Homogenized tissue samples were used for bacterial isolations by direct plating or after enrichment, and for qPCR assay for detection and quantification of *F. necrophorum*.

### Isolations of *F. necrophorum, T. pyogenes, and S. enterica*

Swabs from liver abscesses and homogenized ruminal and colonic tissues were used to inoculate three plates of sheep blood agar (Remel Inc., Lenexa, KS) for the isolation of *F. necrophorum* and *T. pyogenes*, and two plates of Hektoen-Enteric (HE) agar (Beckton, Dickinson and Company, Sparks, MD) for the isolation of *S. enterica*. One blood agar plate (for *F. necrophorum* isolation) and one HE plate (for *Salmonella* isolation) were incubated at 37°C in an anaerobic glove box (Thermo Fisher Scientific Inc., Waltham, MA). The second set of blood and HE agar plates was incubated aerobically. The third blood agar plate was placed in a 5% CO_2_ incubator for *T. pyogenes* isolation. For *F. necrophorum and S. enterica* isolations, an enrichment step was included. One milliliter of tissue homogenates was inoculated into pre-reduced peptone yeast extract medium (PY) supplemented with 100 mM lactate or lysine and antibiotics, josamycin at 3 µg/mL, vancomycin at 4 µg/mL, and norfloxacin at 1 µg/mL (PY-La-JVN or PY-Ly-JVN), and incubated at 37°C for 24 h to enrich *F. necrophorum* for isolation and detection (25). Lysine and lactate serve as key anaerobic energy sources for *Fusobacterium* species, supporting their growth and survival, which facilitates selective enrichment from mixed microbial communities. For *S. enterica,* 10 mL of tissue homogenates was inoculated into 90 mL of tetrathionate broth (Beckton, Dickinson and Co.) and incubated at 37°C for 24 h. Subsequently, 100 µL of the enriched sample was inoculated into 10 mL of Rappaport-Vassiliadis broth (Beckton, Dickinson and Co.) and incubated for an additional 24 h at 42°C. If direct plating of tissue samples did not yield isolates of *F. necrophorum* or *S. enterica,* enriched samples were used to streak the blood agar or HE plates for isolation in respective incubation conditions.

### Isolations of *E. coli*, *K. pneumoniae*, *P. aeruginosa*, and *B. fragilis*

Both direct plating and enrichment methods were employed for the isolation of *E. coli*, *K. pneumoniae*, *P. aeruginosa,* and *B. fragilis*. For *E. coli*, swabs were streaked onto MacConkey agar plates (Beckton, Dickinson and Co.) and incubated at 37°C for 24 h. Lactose-fermenting (pink-colored) and non-mucoid colonies were picked and streaked onto blood agar plates for overnight incubation at 37°C. Colonies that tested positive for the spot-indole test were stored for species confirmation. For *K. pneumoniae*, swabs were streaked onto MacConkey agar containing ampicillin at 10 µg/mL (Sigma-Aldrich, St. Louis, MO; [26]) and incubated at 37°C for 24 h. Lactose-fermenting (pink-colored) and mucoid colonies were picked and streaked onto blood agar plates. Indole-negative colonies were stored for species confirmation. For enrichment of *E. coli* and *K. pneumoniae*, 1 mL of tissue homogenates was inoculated into 9 mL *E. coli* broth (Beckton, Dickinson and Co.) and incubated at 40°C for 6 h. Enriched EC broths were used to streak onto MacConkey agar or MacConkey agar with ampicillin for isolation of *E. coli* and *K. pneumoniae*, respectively, if direct plating of tissue homogenates did not yield isolates. For *P. aeruginosa*, tissue homogenate swabs were streaked onto *Pseudomonas* agar base with CN supplements (cetrimide and nalidixic acid; Oxoid, Basingstoke, UK) and incubated at 37°C for 24 h. Green-blue pigmented colonies were picked and streaked onto blood agar plates for species confirmation. Enrichment involved inoculation of 1 mL of homogenate into 9 mL of acetamide broth (HiMedia Laboratories, Thane, Maharashtra, India) and incubation at 37°C for 24 h, and if direct plating did not yield isolates, enriched samples were streaked onto *Pseudomonas* CN agar. For *B. fragilis*, swabs were streaked onto *B. fragilis* selective (BFS) agar containing supplements (cysteine hydrochloride, bile salts, vitamin K, hemin, ferric ammonium citrate, and bromothymol blue) and antibiotics (gentamicin, kanamycin, and novobiocin). Plates were incubated at 37°C for 48–72 h in an anaerobic glove box. Enrichment involved inoculation into anaerobic BFS broth for 24 h at 37°C, followed by streaking onto BFS agar plates. Large yellow colonies with blackening of the surrounding medium were picked and streaked onto blood agar plates for further confirmation (27).

### Species identification of *F. necrophorum, T. pyogenes,* and *S. enterica*

Presumptive *F. necrophorum* colonies, based on the colony morphology on blood agar plates, were inoculated into pre-reduced anaerobically sterilized brain heart infusion broth (7). The species and subspecies of *Fusobacterium* isolates were confirmed by a qPCR assay described below that targeted the promoter region of the *lktA* gene (25). For *T. pyogenes,* presumptive pin-pointed colonies with a narrow zone of β-hemolysis on blood agar plates were selected and species confirmed by conventional PCR that targeted the pyolysin (*plo*) gene (28). *Salmonella* identification and species confirmation were performed by qPCR assay that targeted the *invA* and *pagC* genes (29).

### Species identifications of *E. coli, K. pneumoniae, and P. aeruginosa*

For *E. coli*, indole-positive isolates were subjected to a three-plex qPCR assay targeting three genes, *uidA*, *clpbB*, and *ybbW*. Isolates positive for any one of the three genes were considered as *E. coli* (30). Species confirmation of *K. pneumoniae* was done by subjecting indole-negative isolates to PCR by targeting 16S-23S internal transcribed spacer genes (31). Presumptive *Pseudomonas* isolates were subjected to genus- and species-specific PCR by targeting 16S rDNA signature sequence (32). The species-confirmed bacterial isolates were stored in cryoprotective beads at −80°C (Cryocare, Key Scientific Products, Round Rock, TX).

### qPCR assay to determine prevalence and concentrations of the two subspecies of *F. necrophorum*

#### DNA extraction

The MagMax-96 DNA Multi-sample Kit (Applied Biosystems, Foster City, CA) was used according to the manufacturer’s protocol for the isolation of DNA from liver abscess, ruminal, and colonic epithelial tissue homogenates for use in the qPCR assay. The DNA extracted from enriched liver abscess, ruminal epithelial, and colonic epithelial samples was purified using the GeneClean Turbo Kit (MP Biomedicals, Solon, OH), following the manufacturer’s protocol for qPCR analysis.

#### QPCR assay

A qPCR assay that targeted the promoter region of the leukotoxin gene, *lktA-n* and *lktA-f,* for subsp. *necrophorum* and subsp. *funduliforme,* respectively, was used to detect and quantify the two subspecies of *F. necrophorum* (25). The assay running conditions were as follows: 95°C for 5 min, followed by 45 cycles of 95°C for 15 s and 60°C for 40 s, using the BioRad CFX96 Real-Time System (BioRad, Hercules, CA). If tissue homogenates were negative for *F. necrophorum,* samples enriched in PY-La-JVN or PY-Ly-JVN were tested by qPCR to determine presence or absence of the two subspecies.

### Statistical analysis

Data analysis was performed in R (version 4.5.0). A generalized linear mixed-effects model with a binomial distribution and logit link function was used to analyze prevalence data of bacterial species, to compare the detection methods (culture vs qPCR) and to evaluate tissue-specific prevalence (liver abscesses vs rumen vs colon; rumen vs colon). Models incorporated fixed effects for detection method or tissue type, while accounting for feedlot-level clustering through random intercepts. Significance was determined using likelihood ratio tests. Fusobacterial concentration data analysis addressed zero-inflation by focusing exclusively on quantifiable (non-zero) samples. Concentrations (CFU/g) were log_10_-transformed and analyzed using linear mixed-effects models (LMMs) with tissue type as a fixed effect and feedlot as a random intercept. Parameter estimation employed Restricted Maximum Likelihood, with likelihood ratio tests evaluating fixed effects. In addition, co-occurrence of *F. necrophorum, T. pyogenes,* and *S. enterica* between liver abscesses and rumen or liver abscesses and colon was assessed by two distinct approaches: within-tissue species associations were analyzed using phi coefficients and the cooccur R package (10,000 permutations), which tested for non-random species pairs in liver, rumen, and colon samples. Between-tissue co-occurrence (rumen-liver or colon-liver) was evaluated through Fisher’s exact tests, where co-occurrence rates were calculated as the proportion of gut-positive cases that were also liver-positive. The initial model included animal-level nesting within feedlot as a random effect, but this term was excluded due to zero variance estimation. Standard errors for tissue means were derived from the estimated marginal means of LMMs.

## RESULTS

### Prevalence of *F. necrophorum* in liver abscesses

Prevalence of *F. necrophorum* in purulent materials of liver abscesses, based on culture, qPCR methods, and by either method, is shown in Table 1. *Fusobacterium necrophorum*, either subsp. *necrophorum* or subsp. *funduliforme,* was detected in all 96 samples by both culture or qPCR method (Table 1). Prevalence of both subspecies together was higher (*P =* 0.001*)* by qPCR method compared to the culture method (25% vs 8.3%). Prevalence of subsp. *necrophorum* by culture method was higher (*P =* 0.001) than the qPCR method (86.5% vs 74%). Total prevalence of subsp. *necrophorum* or subsp. *funduliforme,* which included detection before and after enrichment*,* was similar between the two methods of detection. Seventy-five of 96 (78.1%) liver abscesses contained subsp. *necrophorum* alone, without the subsp. *funduliforme*, and in contrast, only 13 liver abscesses contained subsp. *funduliforme* alone. The culture method yielded the two subspecies of *F. necrophorum* by direct plating of the sample*,* without the need for enrichment. However, qPCR identified a few more samples as positive for the two subspecies after enrichment in lactate or lysine broth.

**TABLE 1:** Culture- and qPCR-based prevalence of the two subspecies of *Fusobacterium necrophorum* in liver abscesses of feedlot cattle fed finishing diets without tylosin

Bacterial species	No. of samples positive (%)			*P*-value, culture vs qPCR
	Culture method	qPCR	Culture or qPCR	
No. of samples cultured	96	96	96	
Subsp. *necrophorum* or *funduliforme* (%)	96 (100)	96 (100)	96 (100)	–^*c*^
Subsp. *necrophorum* and *funduliforme* (%)	8 (8.3)	24 (25.0)	29 (30.2)	0.001
Subsp. *necrophorum*				
Before enrichment of homogenized tissues (%)	83 (86.5)	71 (74)	85 (88.5)	0.002
After enrichment of homogenized tissues in PY-La-JVN^*a*^ broth	0	2 (2.1)	2 (2.1)	–
After enrichment of homogenized tissues in PY-Ly-JVN^*b*^ broth	0	10 (10.4)	10 (10.4)	–
*Necrophorum* without *funduliforme* (%)	75 (78.1)	57 (59.4)	55 (57.3)	0.001
*Necrophorum* with or without *funduliforme* (%)	83 (86.5)	81 (84.4)	96 (100.0)	0.5
Subsp. *funduliforme*				
Before enrichment of homogenized tissues (%)	21 (21.9)	23 (24.0)	26 (27.1)	0.43
After enrichment of homogenized tissues in PY-La-JVN^*a*^ broth	1 (1)	13 (13.5)	14 (14.6)	–
After enrichment of homogenized tissues in PY-Ly-JVN^*b*^ broth	1 (1)	8 (8.3)	9 (9.4)	–
*Funduliforme* without *necrophorum* (%)	13 (13.5)	14 (14.6)	9 (9.4)	0.75
*Funduliforme* with or *necrophorum* (%)	23 (24)	39 (49.6)	41 (42.7)	0.001

^*a*^ Peptone yeast extract with lactate hydrochloride (100 mM) and josamycin (3 µg/mL), vancomycin (4 µg/mL), and norfloxacin (1 µg/mL) antibiotics.

^*b*^ Peptone yeast extract with lysine hydrochloride (100 mM) and josamycin (3 µg/mL), vancomycin (4 µg/mL), and norfloxacin (1 µg/mL) antibiotics.

^*c*^ "–" indicates *P* < 0.05.

### Prevalence of *F. necrophorum* ruminal epithelial and colonic epithelial tissues

Overall, the prevalence of either or both subspecies in ruminal and colonic epithelial tissues was higher (*P* < 0.001) by the qPCR method than by the culture method (Table 2). Prevalence of either of the two subspecies was higher in ruminal epithelial tissue compared to the colonic epithelial tissue (90.6% vs 69.8%). However, prevalence by the culture method was not different between the two epithelial tissues (44.8% vs 43.8). The total prevalence of subsp. *necrophorum,* which included detection before and after enrichment, was low in ruminal compared to the colonic epithelial tissue, but the difference was significant only by the culture method of detection (*P* = 0.004). In contrast, the total prevalence of subsp. *funduliforme* was higher in ruminal epithelial tissue than colonic epithelial tissue, but the difference was significant only by the qPCR method. Detection of either of the two subspecies by the qPCR assay was higher after enrichment than before in either lactate- or lysine-based enrichment broth (Table 2).

**TABLE 2:** Culture- and qPCR-based prevalence of the two subspecies of *Fusobacterium necrophorum* in ruminal epithelial and colonic tissues of feedlot cattle fed finishing diets without tylosin

Bacterial species	No. of samples positive (%)						*P*-value, rumen vs. colon	
	Ruminal epithelial tissues		Culture vs qPCR	Colonic epithelial tissues		Culture vs qPCR		
	Culture method	qPCR		Culture method	qPCR		Culture	qPCR
No. of samples cultured	96	96		96	96			
Subsp. *necrophorum* or *funduliforme* (%)	43 (44.8)	87 (90.6)	<0.001	42 (43.8)	69.8 (44.8)	<0.001	0.93	<0.001
Subsp. *necrophorum* and *funduliforme* (%)	3 (3.1)	25 (26.0)	<0.001	9 (9.4)	21 (21.9)	0.01	0.03	0.70
Subsp. *necrophorum*								
Before enrichment of homogenized tissues (%)	6 (6.3)	3 (3.1)	0.12	19 (19.8)	1 (1.0)		0.004	1.0
After enrichment of homogenized tissues in PY-La-JVN^*a*^ broth	0	18 (18.8)	–^*c*^	0	4 (4.2)	1.0	NA^*d*^	0.004
After enrichment of homogenized tissues in PY-Ly-JVN^*b*^ broth	0	11 (11.5)	–	0	24 (25)	0.9	NA	0
*Necrophorum* without *funduliforme* (%)	3 (3.1)	0	–	10 (10.4)	7 (7.3)	0.45	0.08	–
*Necrophorum* with or without *funduliforme* (%)	6 (6.3)	25 (26.0)	<0.001	19 (19.8)	28 (29.2)	0.11	0.004	0.43
Subsp. *funduliforme*								
Before enrichment of homogenized tissues (%)	40 (41.7)	8 (8.3)	<0.001	32 (33.3)	3 (3.1)	0.001	0.31	1.0
After enrichment of homogenized tissues in PY-La-JVN^*a*^ broth	1 (1)	77 (80.2)	<0.001	0	29 (30.2)	–	0.99	<0.001
After enrichment of homogenized tissues in PY-Ly-JVN^*b*^ broth	0	64 (66.7)	–	0	2 (2.1)	–	NA	<0.001
*fFunduliforme* without *necrophorum* (%)	37 (38.5)	62 (64.6)	<0.001	23 (24.0)	39 (40.6)	–	0.03	<0.001
*Funduliforme* with or *necrophorum* (%)	41 (42.7)	87 (90.6)	<0.001	32 (33.3)	60 (62.5)	<0.001	0.24	<0.001

^*a*^ Peptone yeast extract with lactate hydrochloride (100 mM) and josamycin (3 µg/mL), vancomycin (4 µg/mL), and norfloxacin (1 µg/mL) antibiotics.

^*b*^ Peptone yeast extract with lysine hydrochloride (100 mM) and josamycin (3 µg/mL), vancomycin (4 µg/mL), and norfloxacin (1 µg/mL) antibiotics.

^*c*^ "–" indicates *P* < 0.05.

^*d*^ NA, no *P*-value.

### Concentrations of *F. necrophorum* in liver abscesses, ruminal epithelial, and colonic epithelial tissues

**Fig 1:**
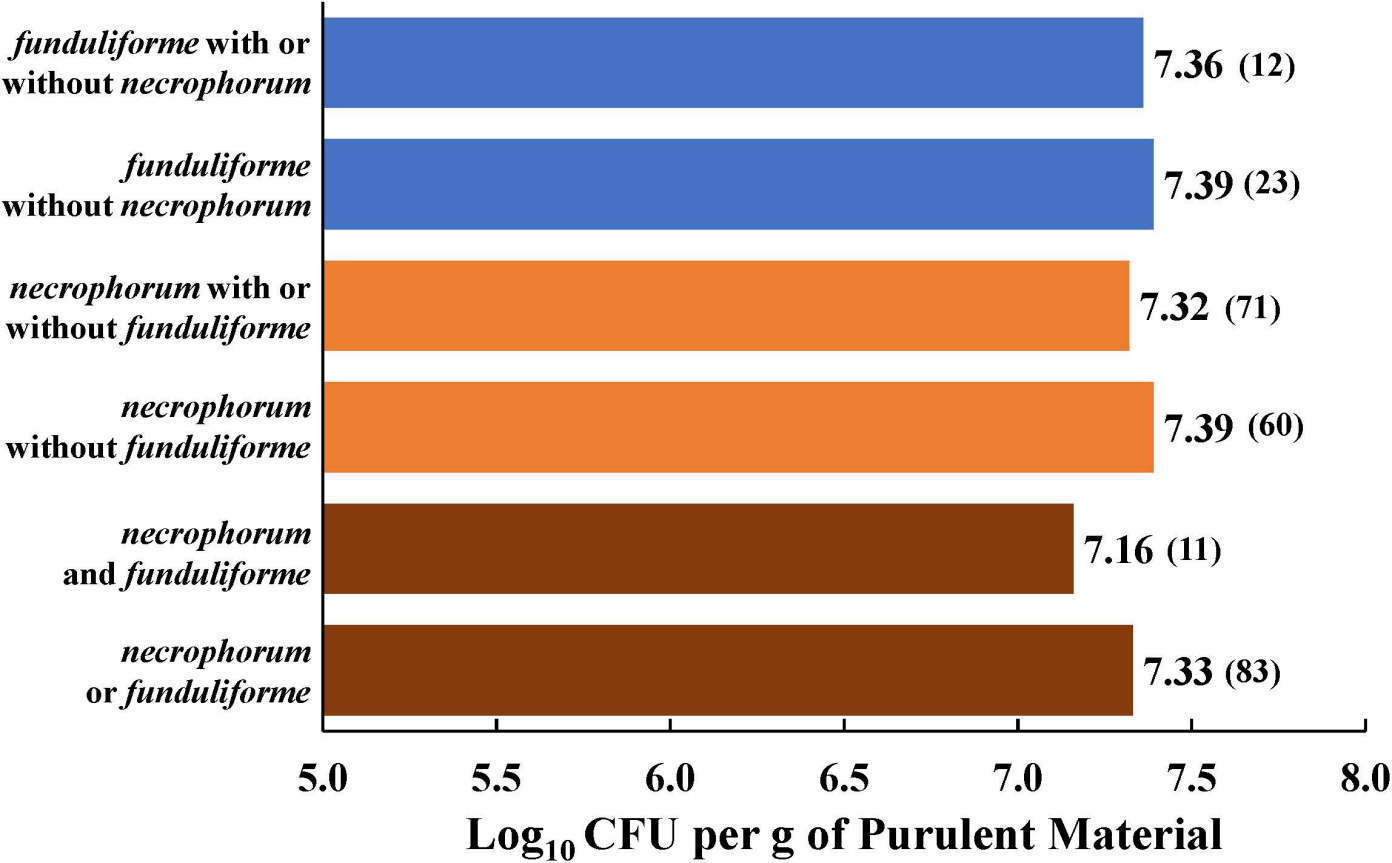
Mean log concentrations (non-zero) of the two subspecies of *Fusobacterium necrophorum,* subsp. *necrophorum* and subsp. *funduliforme,* in liver abscesses of feedlot cattle fed finishing diets without tylosin. Numbers in parentheses are the number of samples that were quantifiable (non-zero).

**Fig 2:**
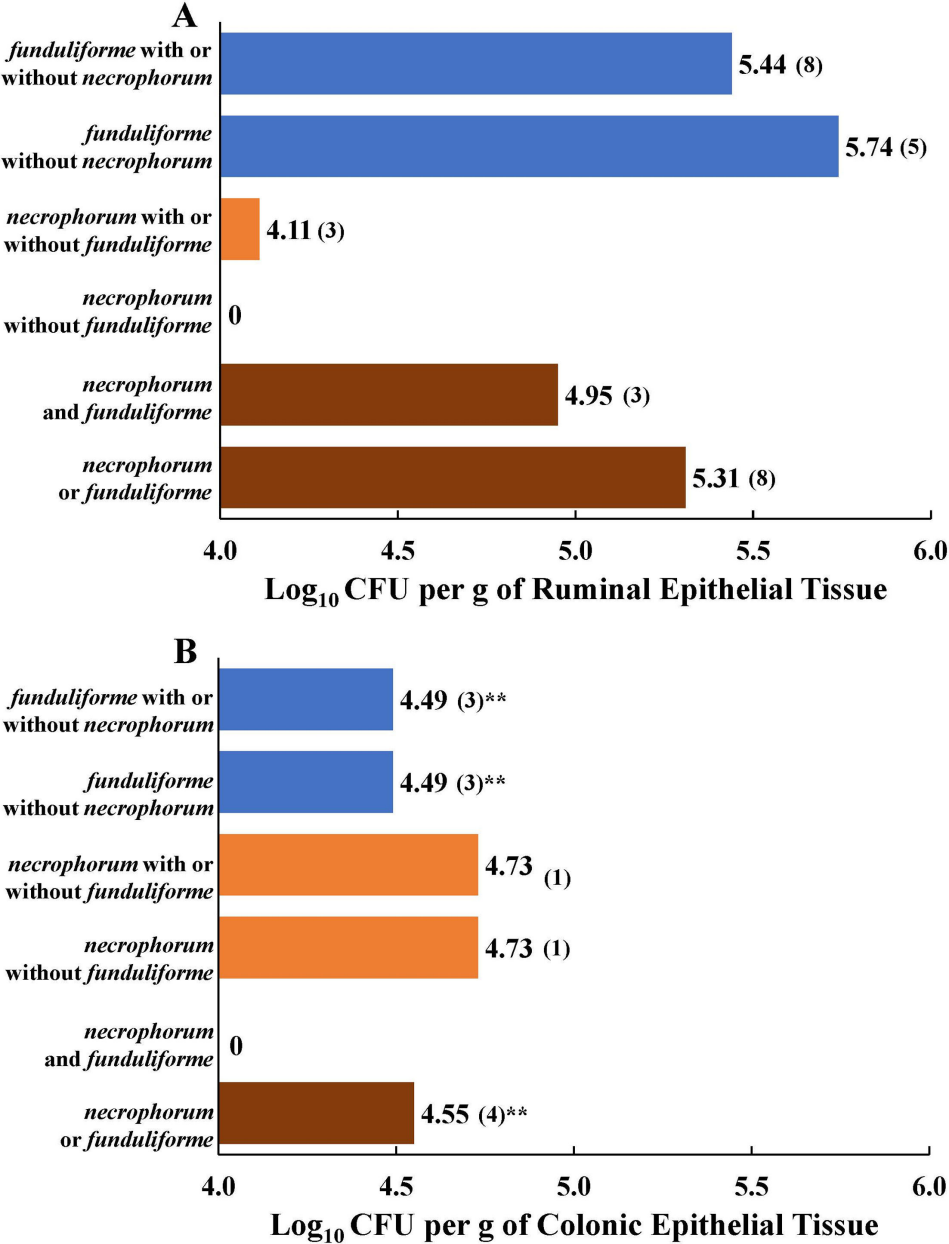
Mean log concentrations (non-zero) of the two subspecies of *Fusobacterium necrophorum,* subsp. *necrophorum* and subsp. *funduliforme,* in ruminal (**A**) and colonic epithelial tissues (**B**) of feedlot cattle fed finishing diets without tylosin. Numbers in parentheses are the number of samples that were quantifiable (non-zero). *Different from concentrations in ruminal epithelial tissue at *P <* 0.05; **Different from concentrations in ruminal epithelial tissue at *P <* 0.01.

The concentrations of the two subspecies in purulent materials of liver abscesses were between 7.0 and 7.5 log_10_ CFU per gram (Fig. 1), and concentrations were similar for the two subspecies when present individually or together. Although all 96 samples were positive for either of the two subspecies, only 83 samples (86%) had quantifiable concentrations, and 60 of those had only subsp. *necrophorum* and only 12 had subsp. *funduliforme,* without the other subspecies. In contrast to liver abscesses, fewer ruminal and colonic epithelial tissues had quantifiable concentrations. Concentrations of either of the two subspecies were in the 4.0 to 5.5 log_10_ CFU range in ruminal epithelial tissues (Fig. 2A) and 4.5 to 5.0 log_10_ CFU range in colonic epithelial tissues (Fig. 2B). The mean concentrations of either of the two subspecies or subsp. *funduliforme* alone or with subsp. *necrophorum* were significantly higher in ruminal epithelial than in colonic epithelial tissues (Fig. 2A and B).

### Isolations of *T. pyogenes* and *S. enterica* in liver abscesses and ruminal and colonic epithelial tissues

*Trueperella pyogenes* was isolated from 34 of 96 liver abscess samples cultured (35.4%), 11 ruminal epithelial tissue samples (11.4%), and was not isolated from any of the colonic epithelial tissues (*P* < 0.001) (Table 3). Only six liver abscess samples (6.3%) yielded *S. enterica*, and none of them was isolated by direct plating of the purulent materials, but were isolated after enrichment. Similar to liver abscesses, only a few ruminal and colonic epithelial tissues yielded *S. enterica,* and more samples yielded the isolates after enrichment than before. The occurrence of *T. pyogenes* and/or *S. enterica* with either of the two subspecies of *F. necrophorum* is shown in Table 3. Only 6 of the 96 abscesses cultured had all three species present. Only one of the ruminal epithelial tissues had co-occurrence of all three species, although a few ruminal epithelial tissues had *F. necrophorum* with *T. pyogenes* or *S. enterica.* Colonic epithelial tissues had co-occurrence of *necrophorum* with *S. enterica* but not with *T. pyogenes.*

**TABLE 3:** Culture-based prevalence of *Trueperella pyogenes* and *Salmonella enterica* individually or in association with *Fusobacterium necrophorum* in liver abscesses and ruminal and colonic epithelial tissues of feedlot cattle fed finishing diets without tylosin

Bacterial species	No. of samples positive (%)			*P*-value
	Liver abscesses	Ruminal epithelial tissues	Colonic epithelial tissues	
No. of samples cultured	96	96	96	
*T. pyogenes*				
Isolation by direct plating of homogenized tissues (%)	34 (35.4)	11 (11.5)	0	0.001
*S. enterica*				
Isolation by direct plating of homogenized tissues (%)	0 (0.0)	2 (2.1)	1 (1.0)	0.21
Isolation by plating homogenized tissues after enrichment^*a*^ (%)	6 (6.3)	2 (2.1)	6 (6.3)	0.09
Total number of isolations (%)	6 (6.3)	4 (4.2)	7 (7.3)	0.48
*F. necrophorum + T. pyogenes*	37 (38.5)	9 (9.4)	9 (9.4)	0.27
*F. necrophorum + S. enterica*	11 (11.5)	5 (5.2)	13 (13.5)	0.005
*F. necrophorum + T. pyogenes + S. enterica*	6 (6.3)	1 (1)	0	0.18

^*a*^ Sample homogenates were enriched in tetrathionate broth at 37°C for 24 h, followed by inoculation into Rappaport-Vassiliadis broth and further incubation at 42°C for 24 h.

### Isolations of *E. coli, K. pneumoniae, P. aeruginosa,* and *B. fragilis* in liver abscesses and ruminal and colonic epithelial tissues

Of the four bacterial species targeted, *E. coli* was the dominant in all three tissue types, and none of the samples was positive for *B. fragilis* (Table 4)*.* In liver abscesses, *E. coli* was isolated from 68 of 96 samples (70.1%), and a majority of isolations were in samples that were enriched in *E. coli* broth. In contrast to liver abscesses, a majority of isolations of *E. coli* from ruminal and colonic epithelial tissues was before enrichment, and the total prevalence, before and after enrichment combined, was 92.7% and 91.6%, respectively. The total prevalence of *E. coli* in ruminal and colonic epithelial tissues was higher than in liver abscesses. A small number (16 or 17) of liver abscesses were positive for both *K. pneumoniae* and *P. aeruginosa.* The prevalence of *K. pneumoniae* was higher (*P <* 0.001) in ruminal epithelial than in liver abscesses or colonic epithelial tissues*. Pseudomonas aeruginosa* was more prevalent in ruminal epithelial tissues than in liver abscesses or colonic epithelial tissues (Table 4).

**TABLE 4:** Isolations of *Escherichia coli*, *Klebsiella pneumoniae, Pseudomonas aeruginosa,* and *Bacteroides fragilis* before and after enrichment in selective media from liver abscesses and ruminal and colonic epithelial tissues of feedlot cattle fed finishing diets without tylosin^*a*^

Bacterial species	No. of samples positive (%)									*P-*value
	Liver abscesses (*n* = 96)			Ruminal epithelial tissue (*n* = 96)			Colonic epithelial tissues (*n* = 96)			
	Before enrichment	After enrichment	Total (%)	Before enrichment	After enrichment	Total (%)	Before enrichment	After enrichment	Total(%)	
*Escherichia coli^b^*	4	64	68^a^ (70.8)	65	24	89^b^ (92.7)	67	21	88b (91.6)	< 0.001
*Klebsiella pneumoniae^b^*	1	16	17^a^ (17.7)	16	32	48^b^ (50.0)	6	9	15^a^ (15.6)	< 0.001
*Pseudomonas aeruginosa^c^*	0	16	16^a^ (16.6)	2	24	26^b^ (27.0)	0	12	12^a^ (12.5)	< 0.001
*Bacteroides fragilis^d^*	0	0	0	0	0	0	0	0	0	-

^*a*^ Total prevalence of each bacterial species with different superscripts differs at *P *< 0.001.

^*b*^ Enrichments included inoculation of homogenates into *E. coli* broth and incubation at 40°C for 6 h.

^*c*^ Enrichment included inoculation of homogenates into acetamide broth and incubation at 37°C for 24 h.

^*d*^ Enrichment included inoculation of homogenates into anaerobic *Bacteroides fragilis* selective broth for 24 h at 37°C.

### Co-occurrence of *F. necrophorum, T. pyogenes, and S. enterica* in liver abscesses and in ruminal and colonic epithelial tissues

*Fusobacterium* subspecies demonstrated distinct patterns in their association with liver abscesses. The subsp. *necrophorum* showed strong co-occurrence with liver abscesses regardless of the gut origin (Table 4). When present in the rumen, it co-occurred in liver abscesses in 83.3% of cases (5/6), and in the colon, the rate was 89.5% (17/19). In contrast, the subsp. *funduliforme* exhibited weaker associations. Only 17.1% of rumen cases (7/41) and 31.2% of colon cases (10/32) co-occurred with liver abscesses. Although colon co-occurrence was numerically higher than the rumen, there were no significant differences between gut sites for both subspecies (*P* = 1.0 for subsp. *necrophorum* and *P* = 0.1745 for subsp. *funduliforme*). No significant difference in co-occurrence rates was observed between ruminal and colonic origins for *F. necrophorum* subsp. *necrophorum* (*P* = 1.0). *Trueperella pyogenes* displayed a strong site-specific pattern. When detected in the rumen, it co-occurred with liver abscesses in 90.9% of cases (10/11). Because *T. pyogenes* was not detected in the colon, the colon-liver co-occurrence was not assessed. *Salmonella enterica* had limited evidence of liver abscess involvement because of low prevalence in all three tissues. Only 50% of rumen cases (1/2) and 66.7% of colon cases (4/6) co-occurred with liver abscesses (Table 5).

**TABLE 5:** Co-occurrence of major bacterial species in liver abscesses, rumen, and colonic epithelial tissues of feedlot cattle fed finishing diets without tylosin

Bacterial species	Liver abscesses and ruminal epithelial tissues	Co-occurrence rate	Liver abscesses and colonic epithelial tissues	Co-occurrence rate^*a*^
*Fusobacterium necrophorum* subsp. *necrophorum*	5/6	0.83	17/19	0.90
*Fusobacterium necrophorum* subsp. *funduliforme*	7/41	0.17	10/32	0.31
*Trueperella pyogenes*	10/11	0.91	0/0	0
*Salmonella enterica*	1/2	0.5	4/6	0.67

^*a*^ Co-occurrence rate was calculated as the proportion of number of samples where the species was present in both gut tissue and liver abscesses divided by number of samples where the species was present in gut tissues.

## DISCUSSION

A major impetus for this study was to revisit the etiology and pathogenesis of liver abscesses because vaccines that have been developed based on *F. necrophorum and T. pyogenes* antigens (bacterins and or leukotoxin-based) have been ineffectual to marginally effective (33, 34). Moreover, tylosin, the most commonly used in-feed antimicrobial agent, is not 100% effective in reducing the incidence of liver abscesses (35, 36). A further delineation of the etiology and assessment of the hindgut as another source of liver abscess pathogens may lead to identifications of novel targets, pathogens, for the development of more effective intervention strategies. Almost all studies published on the etiology of liver abscesses have focused on the three pathogens, *F. necrophorum, T. pyogenes,* and *S. enterica,* involved in liver abscesses, but have reported occurrence of lesser-known pathogens as sporadic (10, 11, 33). In a study on the culture-based bacterial analysis of liver abscesses of crossbred beef cattle and Holstein steers fed finishing diets with or without tylosin, in addition to *Fusobacterium, Trueperella,* and *Salmonella,* two bacterial species, *E. coli* (32.4%) and *K. pneumoniae* (14.8%), were isolated more frequently than others (6)*.* The isolations were based on direct plating of purulent materials on general-purpose media and did not use any enrichment or selective media for isolations of *E. coli* and *K. pneumoniae.* The culture-independent methods that have analyzed bacterial community composition of liver abscesses have indicated that Pseudomonadota and Bacteroidota were the two dominant phyla, next only to the phylum Fusobacteriota, and *Bacteroides* as the second most dominant genus, next to *Fusobacterium* (12–15, 37, 38). Generally, microbiome studies have indicated that only a small proportion of liver abscesses are dominated by organisms other than the *Fusobacterium*. Pinnell et al. (15) have reported that of the 259 liver abscesses analyzed by 16S amplicon sequencing, 61 (23.6%) abscesses were dominated by *Bacteroides* and *Porphyromonas*. A recent report on 16S amplicon-based sequence analysis of 30 liver abscesses from cattle fed tylosin indicated that Fusobacteriota and Bacteroidota accounted for 85.9% and 12.6% of the prominent taxa (14).

This is the first study that utilized selective enrichment and isolation methods to determine the prevalence of *E. coli, K. pneumoniae, P. aeruginosa*, and *B. fragilis* in liver abscesses. Of the three species that belong to the phylum Pseudomonodata (formerly Proteobacteria), *E. coli* was the most dominant species detected in liver abscesses (70.8%), next only to the prevalence of *F. necrophorum* (100%). The species, a common gut bacterium, is well known to thrive across the gastrointestinal tract because of its adaptability to grow at different pH and even under anaerobic conditions (39). However, almost all isolations of *E. coli* were from liver abscesses that were subjected to an enrichment step, which suggests that the concentrations were too low; therefore, not likely to be contributing to the infection. Interestingly, in ruminal and colonic epithelial tissues, a majority of *E. coli* isolations was before enrichment, suggesting the concentrations were much higher than in liver abscesses. The high concentrations in ruminal and colonic tissues suggest that *E. coli* could be contributing to inflammation and infection of the ruminal epithelial tissues, but confirmation would require assessment of virulence potential by whole-genome sequence analysis. The detection of the other two species, *K. pneumoniae* and *P. aeruginosa,* was primarily after enrichment in all three samples, suggesting prevalence of low concentrations, therefore not likely to be contributing to rumenitis or liver abscesses. Interestingly, the association of *E. coli* with the two liver abscess pathogens, *F. necrophorum* and *T. pyogenes,* has also been observed in one other infection, metritis, in dairy cows (40–42). It is suggested that early uterine contamination with *E. coli* leads to subsequent infection by *F. necrophorum* and *T. pyogenes* (43, 44). It is tempting to speculate that a similar sequence of invasion can happen with liver abscesses because *E. coli* has been shown to be a dominant organism in healthy livers (9).

The reason for including *B. fragilis* in our study was that the species is one of the dominant anaerobes in the hindgut of animals and humans (45) and is the most virulent of the *Bacteroides* species, hence frequently isolated from intra-abdominal and liver abscesses of humans (46). The capsule of *B. fragilis,* composed of two distinct high-molecular-weight polysaccharides, is responsible for inducing abscesses (47). Although selective enrichment and isolation media were used, none of the 96 liver abscess samples yielded *B. fragilis,* and the absence is in agreement with a recent study that utilized a combination of culture-independent and culture-dependent methods to isolate and identify species of *Bacteroides* associated with liver abscesses (37). In the study, none of the liver abscess samples was positive for *B. fragilis*; instead, *B. pyogenes* and a novel species, *B. purulensis*, were identified (37).

Liver abscesses have long been described as polymicrobial infections that are primarily composed of gram-negative bacteria (10, 11, 33). Almost all culture-based studies have indicated that *F. necrophorum*, specifically the subsp. *necrophorum,* is the primary etiologic agent, and the frequency of isolations has ranged from 85 to 100% of liver abscesses (5, 10). In the current study, all 96 liver abscess samples yielded either of the two subspecies, and the proportions of the distribution of the two subspecies were 86.5% and 24% for subsp. *necrophorum* and subsp. *funduliforme*, respectively. In a summary of six studies reported by Amachawadi and Nagaraja (5), which involved analyses of 846 liver abscesses, the mean prevalence of the subsp. *necrophorum* and subsp. *funduliforme* was 89% and 23.5%, respectively. The dominance of the subsp. *necrophorum* is generally attributed to its higher virulence because of increased production of virulence factors, specifically the leukotoxin production (48, 49). In a recent report of liver abscesses of beef-on-dairy cattle, in the no-tylosin-fed, negative control group, 94.7% of abscesses cultured were positive for subsp. *necrophorum* compared to 47.4% for subsp. *funduliforme* (50). In the current study, 8% and 25% of abscesses had both subspecies by culture and qPCR methods, respectively.

In the culture method, although samples were enriched in lactate- or lysine-based selective medium, almost all the isolations were from direct plating of the purulent material, suggesting a relatively high concentration of *F. necrophorum* in purulent materials*.* However, the qPCR assay identified some samples as positive for *F. necrophorum* only after enrichment. Although the prevalence of subsp. *necrophorum* was similar between the two detection methods, the qPCR assay detected a higher proportion of liver abscesses as positive for subsp. *funduliforme* than the culture method. Concentrations of the two subspecies in liver abscesses were in the log_10_ 7 to 7.5 CFU per gram range, and the concentrations were similar, regardless of the presence of the two subspecies together or individually. The high concentrations are indicative of their active growth and contributions to the development of the abscesses. This is the second study to quantify the *F. necrophorum,* and the concentrations are in agreement with the report in liver abscesses of beef-on-dairy cattle (50).

Of the two subspecies of *F. necrophorum,* the prevalence of subsp. *funduliforme* was higher in both ruminal and colonic epithelial tissues than the subsp. *necrophorum.* In both tissues, the prevalence was much higher by the qPCR assay than by the culture method, likely because both tissues are colonized by dense populations of epimural bacteria (51), thus making it relatively difficult to detect by culture method. Only a few tissue samples had quantifiable concentrations of either of the two subspecies, and the concentrations were one log higher in ruminal epithelial than in colonic epithelial tissue. This is the first study to provide prevalence and concentrations of the two subspecies in colonic epithelial tissue, which suggests that the colon is likely to be another gut region from which *F. necrophorum* originates and travels to the liver via portal blood.

The central dogma on the role of the rumen in the pathogenesis of liver abscesses is based on the observations of a positive association between the occurrence of liver abscesses and ruminal pathology associated with ruminal acidosis (16–18). In addition, *F. necrophorum* is a normal inhabitant of the rumen; therefore, it is a logical source of infection of the liver (52). In cattle fed high-grain diets, there is a flow of a certain amount of starch into the hindgut, where it gets fermented to volatile fatty acids (VFA), possibly lactic acid, resulting in varying degrees of acidosis. There is recognition that hindgut acidosis is a component of the overall ruminal and gut acidosis syndrome, and dysbiotic changes, such as bacterial diversity, accumulation of metabolites, and toxic products are similar to that of the rumen (53–55). Another factor that may contribute to the importance of hindgut acidosis is the histological differences in the epithelia between the rumen and hindgut. Hindgut epithelium, a monolayer of columnar epithelial cells, in contrast to stratified squamous epithelium of the rumen, is likely to be more susceptible to disruption of the protective barrier function and bacterial translocation. In order to assess the potential role of the hindgut as a source of liver abscess pathogens, colonic epithelial tissues were included in the study. Liver abscesses were matched with ruminal epithelial and colonic epithelial tissue so that the prevalence of major and minor pathogens and concentrations of *F. necrophorum* in liver abscesses could be compared to ruminal and colonic epithelial tissues. Cattle that were sampled in the study originated from feedlots, which did not include in-feed tylosin. The justification was to avoid potential effects of tylosin on the bacterial community composition, particularly in the gut epithelial tissues. Interestingly, colonic epithelial tissues yielded more subsp. *necrophorum* isolates (19/96) than the ruminal epithelial tissue (6/96). The co-occurrence of the two subspecies between liver abscesses and ruminal and colonic epithelial tissues indicated a higher rate in colonic epithelial tissue (89.5%) than in ruminal epithelial tissue (83.3%), although the difference was not significant, likely because only a few cattle had co-occurrence between the two sites. The lack of significance (*P* = 1.0) should be interpreted with caution because of the small sample size.

Although the data suggest that the hindgut could be a source of *F. necrophorum* that reaches the liver, confirmation would require whole-genome sequencing analysis to ascertain clonal identity. The genetic identity of *F. necrophorum* strains between liver abscesses and ruminal epithelial tissues has been shown by DNA sequencing of rRNA genes (19). Of the two secondary pathogens associated with liver abscesses, *T. pyogenes* was isolated only from ruminal epithelial tissue, but not from the colonic epithelial tissue. This was an unexpected finding, and the absence of *T. pyogenes* in colonic epithelial tissue is difficult to explain. Because we did not use a PCR assay and an enrichment step, it is difficult to conclude the true absence in the tissue. In a serial slaughter study of feedlot cattle that evaluated corn stalks and tylosin inclusions in the diet, *T. pyogenes* was isolated from the ruminal and ileal epithelial tissues, but not from colonic epithelial tissue (24). In contrast to *T. pyogenes*, colonic epithelial tissues had higher prevalence of *S. enterica* than the ruminal epithelial tissue, suggesting that the likely source of *Salmonella* in liver abscesses is the hindgut. In a study to determine the link between bacterial communities in liver abscesses and the gut, Pinnell et al. (56) observed that the prevalent taxa in liver abscesses not dominated by *Fusobacterium* were more abundant in the ileum and colon than the rumen.

### Conclusion

The results of our study, based on culture and qPCR methods, reaffirm that liver abscesses in cattle are polymicrobial infections, and *F. necrophorum,* specifically the subsp. *necrophorum,* is the most dominant species prevalent and therefore likely to be the primary etiologic agent. Of the three lesser-known pathogens that were selectively targeted for isolation, *E. coli* was the second most dominant species, next to *F. necrophorum* subsp. *necrophorum*. However, a majority of the isolations of *E. coli* were after enrichment of the sample, suggesting that the concentration was too low to be contributing to the abscess development. The high prevalence of *E. coli* in ruminal and colonic epithelial tissues, possibly at high concentration because the majority of the isolations were before enrichment, suggests that it may be contributing to rumenitis and disruption of ruminal epithelial integrity, thus creating an entry point for other pathogens, such as *Fusobacterium necrophorum* and *T. pyogenes*, to translocate into the bloodstream. A major finding of this study is the prevalence and isolations of both subspecies of *F. necrophorum* in colonic epithelial tissues*,* and the higher frequency of subsp. *necrophorum* isolation from colonic than from ruminal epithelial tissues, which suggests that the hindgut, besides the rumen, serves as a source of bacterial pathogens, particularly *F. necrophorum* and *S. enterica,* that reach the liver. This finding suggests that the hindgut should also be targeted for effective intervention to reduce the incidence of liver abscesses. Tylosin, the most commonly used feed additive in the industry, may not reach the hindgut in active form to exert its antifusobacterial activity. Therefore, it is tempting to surmise that the reason why tylosin is not 100% effective in preventing liver abscesses is because some pathogens are originating from the hindgut. Additional research is needed to confirm the clonal identity of *F. necrophorum* from liver abscesses with that of colonic epithelial tissue and to assess the extent of hindgut contribution to the liver abscess development.

## Data Availability

All the data generated are presented in tables in the manuscript.
